# Comparative analysis of the human microbiome from four different regions of China and machine learning-based geographical inference

**DOI:** 10.1128/msphere.00672-24

**Published:** 2024-12-19

**Authors:** Yinlei Lei, Min Li, Han Zhang, Yu Deng, Xinyu Dong, Pengyu Chen, Ye Li, Suhua Zhang, Chengtao Li, Shouyu Wang, Ruiyang Tao

**Affiliations:** 1 Shanghai Key Laboratory of Forensic Medicine, Shanghai Forensic Service Platform, Academy of Forensic Sciences, Key Laboratory of Forensic Science, Ministry of Justice, Shanghai, China; 2 Department of Forensic Medicine, Zunyi Medical University 66367 , Zunyi, China; 3 School of Clinical and Basic Medical Sciences, Shandong First Medical University & Shandong Academy of Medical Sciences, Jinan, China; 4 Institute of Forensic Science, Fudan University, Shanghai, China; 5 Department of Forensic Medicine, Guizhou Medical University 74628 , Guiyang, China; 6 Minhang Branch of Shanghai Public Security Bureau, Shanghai, China; 7 Department of Forensic Medicine, School of Basic Medical Sciences, Xinjiang Medical University, Urumqi, China; 8 Department of Forensic Medicine, Shanghai Medical College, Fudan University, Shanghai, China; University of South Africa, Florida, Johannesburg, Gauteng, South Africa

**Keywords:** human microbiome, 16S rRNA, geographic regions, machine learning

## Abstract

The human microbiome, the community of microorganisms that reside on and inside the human body, is critically important for health and disease. However, it is influenced by various factors and may vary among individuals residing in distinct geographic regions. In this study, 220 samples, consisting of sterile swabs from palmar skin and oral and nasal cavities were collected from Chinese Han individuals living in Shanghai, Chifeng, Kunming, and Urumqi, representing the geographic regions of east, northeast, southwest, and northwest China. The full-length 16S rRNA gene of the microbiota in each sample was sequenced using the PacBio single-molecule real-time sequencing platform, followed by clustering the sequences into operational taxonomic units (OTUs). The analysis revealed significant differences in microbial communities among the four regions. *Cutibacterium* was the most abundant bacterium in palmar samples from Shanghai and Kunming, *Psychrobacter* in Chifeng samples, and *Psychrobacillus* in Urumqi samples. Additionally, *Streptococcus* and *Staphylococcus* were the dominant bacteria in the oral and nasal cavities. Individuals from the four regions could be distinguished and predicted based on a model constructed using the random forest algorithm, with the predictive effect of palmar microbiota being better than that of oral and nasal cavities. The prediction accuracy using hypervariable regions (V3-V4 and V4-V5) was comparable with that of using the entire 16S rRNA. Overall, our study highlights the distinctiveness of the human microbiome in individuals living in these four regions. Furthermore, the microbiome can serve as a biomarker for geographic origin inference, which has immense application value in forensic science.

**IMPORTANCE:**

Microbial communities in human hosts play a significant role in health and disease, varying in species, quantity, and composition due to factors such as gender, ethnicity, health status, lifestyle, and living environment. The characteristics of microbial composition at various body sites of individuals from different regions remain largely unexplored. This study utilized single-molecule real-time sequencing technology to detect the entire 16S rRNA gene of bacteria residing in the palmar skin, oral, and nasal cavities of Han individuals from four regions in China. The composition and structure of the bacteria at these three body sites were well characterized and found to differ regionally. The results elucidate the differences in bacterial communities colonizing these body sites across different regions and reveal the influence of geographical factors on human bacteria. These findings not only contribute to a deeper understanding of the diversity and geographical distribution of human bacteria but also enrich the microbiome data of the Asian population for further studies.

## INTRODUCTION

Microorganisms, including bacteria, viruses, and fungi, are widely and stably present in the natural environment and the human body, playing a significant role in human health and disease (1–3). However, due to varying environmental factors (such as climate, rainfall, altitude, and soil), hereditary factors, and personal habits, the composition of microbial communities differs across distinct geographical locations (4, 5). Therefore, conducting a human microbiome survey of individuals from different regions is a highly meaningful research endeavor.

Human skin acts as a physical barrier between the human body and the environment, protecting the body from the invasion of foreign pathogens and serving as a vector for microbiota communities (6). It is also a dynamic system, with different properties across skin sites. Palmar skin, which frequently interacts with surroundings through touch, harbors microbial communities susceptible to the influence of environmental factors and personal lifestyles (7). Similarly, oral and nasal cavities, as major gateways to the human body, provide a moist and warm environment where microbial cells reside. These mucosal regions, influenced by various factors such as ingested food and saliva, significantly impact the oral microbiota, which in turn affects human disease evolution and response to therapy (8–10). The nasal cavity, often colonized by facultative microbial cells like Staphylococcus aureus, plays a critical role in respiratory health, as highlighted during the COVID-19 pandemic (11, 12). However, the nasal cavity microbiota remains under-characterized. Therefore, understanding the structure and diversity of microbiota on palmar skin and in the oral and nasal cavities is crucial for studies on human health and disease.

High-throughput DNA sequencing technology has facilitated the investigation of the human microbiome, revealing significant differentiation in bacterial communities across different regions. For instance, Li et al. demonstrated differences in saliva microbiome diversity among populations from various climate zones (8). Escobar et al. found geographic heterogeneity in the gut microbiota of Colombians compared with Americans, Europeans, and Asians, with notable differences in Actinobacteria and Verrucomicrobia (5). Clarke et al. analyzed human stool and oral microbiomes from Barbados, Santiago (Chile), Pretoria (South Africa), and Bangkok (Thailand), revealing detectable geolocation differences that were not influenced by other lifestyle variables (13). Collectively, these studies illustrate the human microbiome composition in populations from different geographic locations, providing new directions for geographic tracing based on the human microbiome. However, current studies on microbial geographic differences are limited, with inadequate geographic coverage of samples. In China, to our knowledge, only the oral microbiota of the people in Chengdu and Tibet, the soil bacteria from several regions, as well as microbiota from shoe soles and shoeprints have been the subject of relevant geographical traceability studies (14–16). Therefore, we aim to conduct studies in several additional cities that are regionally representative of China, along with an investigation of their bacterial characteristics.

Shanghai, situated in the eastern coastal region of China, experiences a warm and humid climate. Chifeng and Urumqi are located in the northern grassland region and the arid northwestern region of China, respectively. Chifeng features a typical grassland climate, whereas Urumqi experiences a dry climate characterized by significant temperature fluctuations, and in both areas, the diet is predominantly centered around meat and dairy products. Kunming, in the southwestern highland region, enjoys a subtropical monsoon climate with consistently mild temperatures throughout the year. These regions roughly represent the eastern, northeastern, northwestern, and southwestern regions of China and display notable differences in climate, dietary habits, and lifestyles. By exploring the microbiome characteristics of Han populations in these regions, new perspectives and directions can be provided for geographic tracing research based on human microbiomes.

Additionally, many studies have focused on sequencing and analyzing hypervariable regions of the bacterial 16S rRNA gene, typically identifying microbes at the genus level (17). For specific information on microbes from distinct regions, higher resolution classification and identification are required. Third-generation sequencing technology, such as that based on the PacBio platform, allows for the reading of long nucleotide sequences spanning the entire length of the 16S rRNA gene through single-molecule real-time (SMRT) sequencing. Circular consensus sequencing (CCS) is employed in SMRT to rectify sequence errors, ensuring the generation of high-fidelity microbiome data at the species level (18, 19). In recent years, full-length 16S rRNA gene sequencing has been shown to provide better resolution for distinguishing members within a studied microbial community (20, 21).

Therefore, in this study, third-generation sequencing was employed to detect the full-length 16S rRNA gene of the microbiota from the palmar skin, oral, and nasal cavities of Chinese Han individuals from Chifeng, Kunming, Shanghai, and Urumqi. The aim was to conduct a comparative analysis of the human microbiome composition and diversity of individuals living in these four regions and characterize variations in the microbiota relative to body sites and inherent inter-region differences. Moreover, the performance of short hypervariable regions (V3-V4 and V4-V5) and full-length 16S rRNA in microbiome investigation and geographical tracing was analyzed and compared *in silico*. This study also enriched the microbiome data of the Asian population.

## RESULTS

Full-length 16S rRNA sequencing of 220 samples yielded a total of 1,577,010 CCS reads, with an average of 7,168 CCS reads per sample. After quality control filtering, 1,554,406 effective CCS reads were retained, with an average length of 1,464 bp (Table S1). The Shannon curve, reflecting the microbial diversity of samples at different sequencing depths, indicated comprehensive coverage of all microbial taxa within these samples, meeting the required analysis standards. Based on these sequences, a total of 61,130 OTUs were identified, encompassing 1,901 species, 731 genera, 285 families, 127 orders, 53 classes, 29 phyla, and 1 kingdom. Specifically, 25,654, 21,281, and 14,195 OTUs were obtained from palmar skin, oral, and nasal samples, respectively. Microbial samples from Shanghai, Chifeng, Kunming, and Urumqi contained 15,089, 20,867, 13,979, and 11,195 OTUs, respectively, with 2,398 OTUs shared among these regions.

### The microbiome diversity of three body sites across four regions

**Fig 1:**
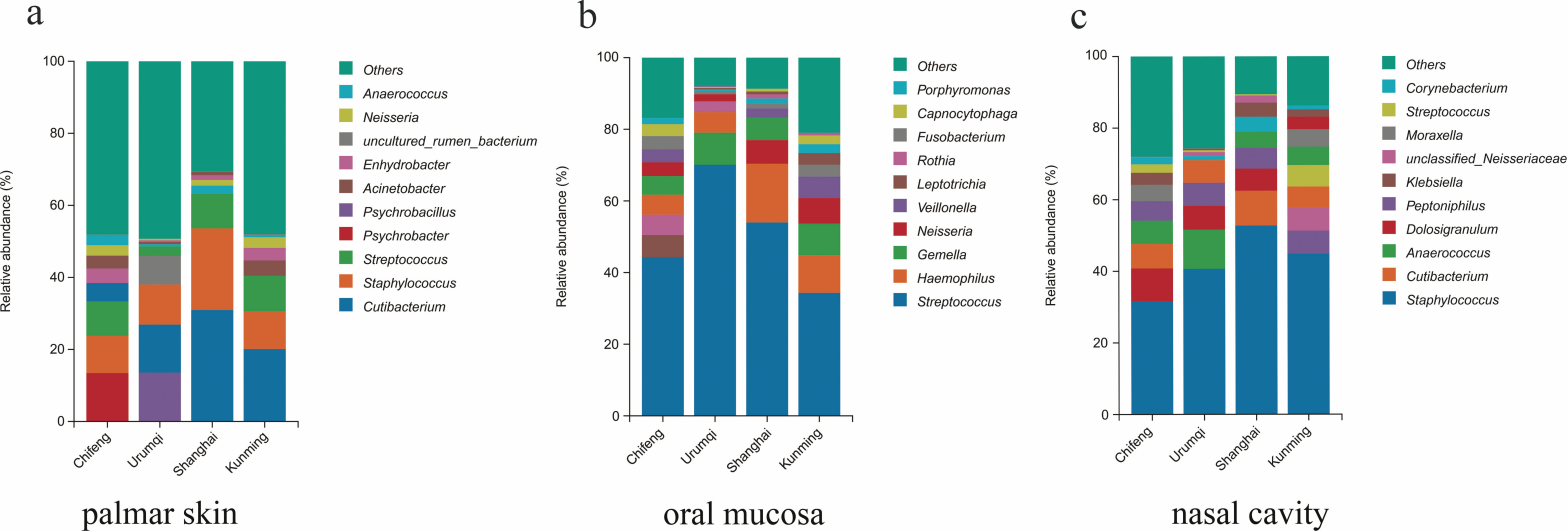
Bacterial community composition of the (**a**) palmar skin, (**b**) oral mucosa, and (**c**) nasal cavity swab samples from four regions at the genus level.

For microbiome composition, the top 10 most abundant bacterial genera of palmar skin, oral, and nasal cavities in each region were selected (Table S2). The bacteria present on the surface of palmar skin primarily included *Cutibacterium*, *Staphylococcus*, *Streptococcus,* and so forth. The bacterium with the highest abundance was *Cutibacterium* in Shanghai (31%) and Kunming (20%), *Psychrobacter* in Chifeng (13%), and *Psychrobacillus* in Urumqi (14%) (Fig. 1a). Oral bacteria were mainly composed of *Streptococcus*, *Haemophilus*, *Gemella*, and *Neisseria*, with *Streptococcus* being the most common bacterium across the four regions (relative abundance from 34% to 70%, Fig. 1b). Nasal bacteria primarily consisted of *Staphylococcus*, *Cutibacterium*, *Anaerococcus*, and *Dolosigranulum*, with *Staphylococcus* being the dominant bacterium in nasal samples from the four regions (relative abundance from 32% to 53%, Fig. 1c).

At the species level, the top 10 abundant bacteria were also screened (Table S3). The most abundant bacteria on the palmar skin were *Psychrobacter sanguinis* (12%) in Chifeng and *Psychrobacillus psychrodurans* (14%) in Urumqi. In Shanghai and Kunming, *Cutibacterium acnes*, *Staphylococcus caprae*, *Staphylococcus epidermidis,* and *Streptococcus mitis* accounted for more than 30% of the palmar bacteria (Fig. S1a). Oral bacteria mainly consisted of *Streptococcus mitis*, *Haemophilus parainfluenzae*, *Staphylococcus pasteuri*, *Gemella haemolysans*, and others, with *Streptococcus mitis* being the most abundant bacterium (average relative abundance of 44%) across the four regions (Fig. S1b). For nasal bacteria, *S. epidermidis*, *Dolosigranulum pigrum*, *Peptoniphilus rhinitidis*, and *Anaerococcus octavius* accounted for more than 50% of the nasal bacteria across the four regions, with *S. epidermidis* being the most abundant bacterium (average relative abundance of 35%) (Fig. S1c).

**Fig 2:**
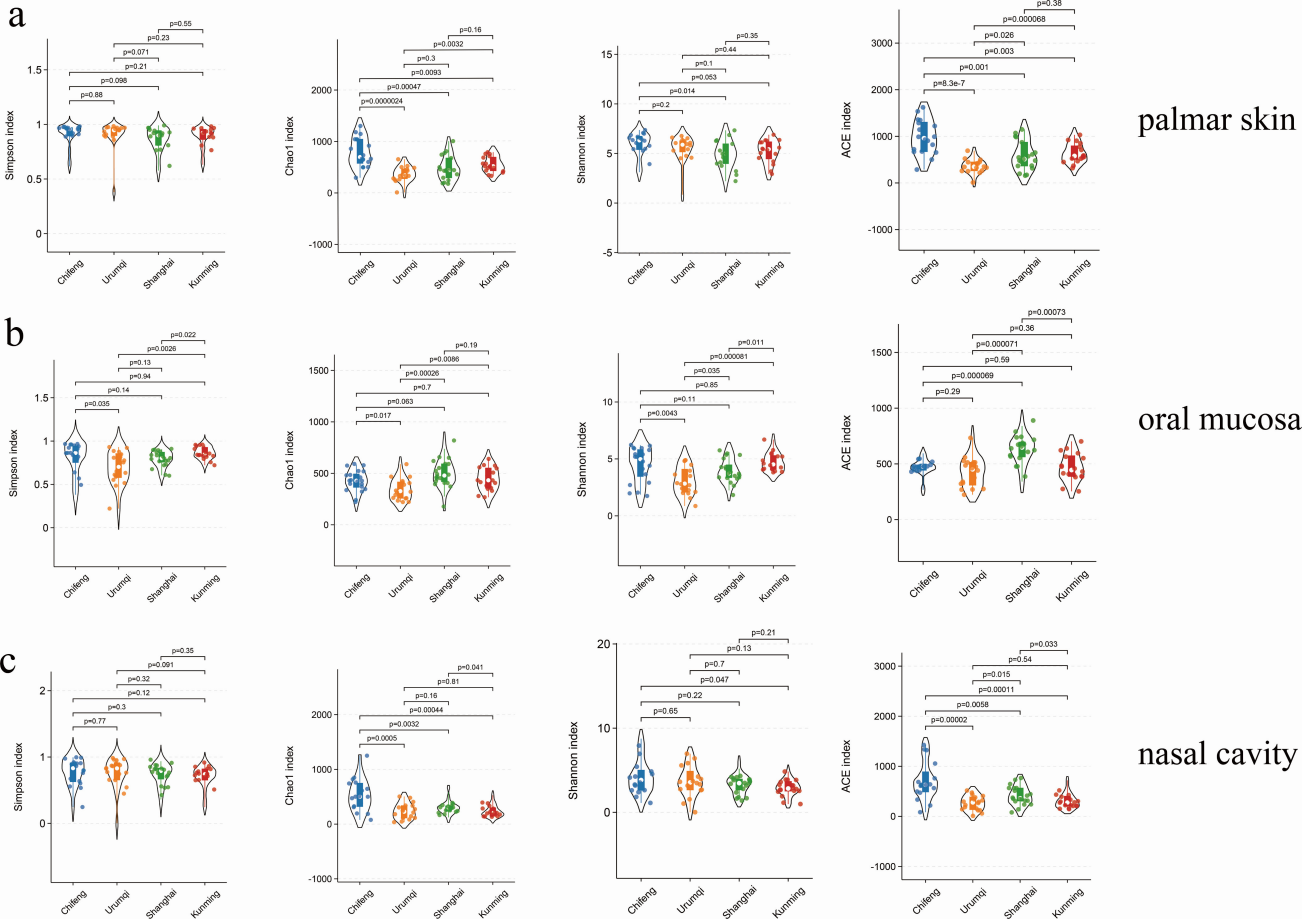
The Simpson, Chao 1, Shannon, and ACE indices of the (**a**) palmar skin, (**b**) oral mucosa, and (**c**) nasal cavity swab samples from four regions (***, *P*＜0.001; **, *P*＜0.01; *, *P*＜0.05).

The indices of Simpson, Chao 1, Shannon, and ACE were used to comprehensively reflect the alpha diversity of the samples, and the Wilcoxon signed-rank test was then used to analyze statistical differences between the groups. For palmar skin samples, the Simpson index values approached one in these regions, with significant differences in bacterial richness observed between most regions except Shanghai and Kunming, as shown by the Chao1 and ACE indices. Additionally, the highest Shannon index was recorded in the Chifeng region, followed by Urumqi, Kunming, and Shanghai (Fig. 2a). Similarly, for oral samples, the Simpson index approached one in four regions, whereas it exhibited greater variability in the Chifeng and Kunming groups. Significant differences in the Chao1 index were observed between Urumqi and other regions. The Shannon index in Chifeng was higher than that of Kunming, Shanghai, and Urumqi groups. Notably, there were significant differences between the Urumqi samples and those from the other regions and between Kunming and Shanghai groups (Fig. 2b). Regarding nasal bacteria, the analysis revealed significant differences in bacterial richness (Chao1 and ACE indices) between Chifeng and other regions. Furthermore, the highest Shannon index was observed in samples from Chifeng, followed by Urumqi, Shanghai, and Kunming groups (Fig. 2c).

### The variations in human microbiome community structures across four regions

**Fig 3:**
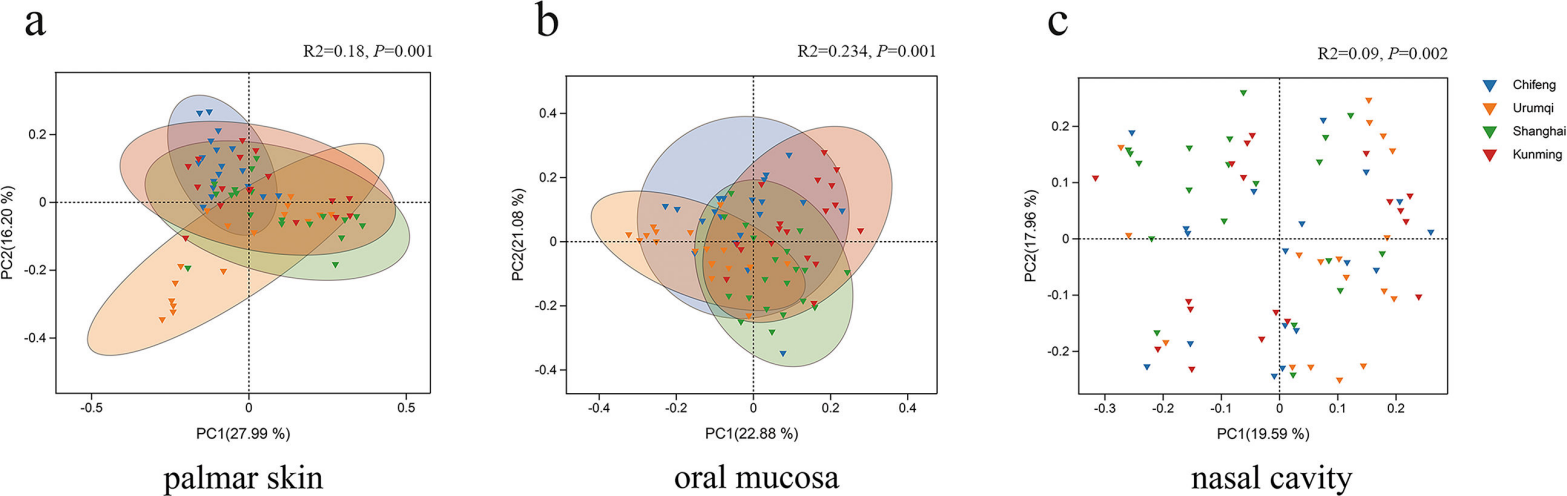
Principal coordinates analysis (PCoA) of the (**a**) palmar skin, (**b**) oral mucosa, and (**c**) nasal cavity swab samples from four regions, based on bacterial OTUs.

Based on the PCoA results using the Weighted_Unifrac distance matrix, bacteria from the palmar skin showed that Chifeng samples clustered together in the second quadrant, Kunming samples were primarily distributed in the first and second quadrants, and Urumqi samples were mainly distributed in the third and fourth quadrants. PERMANOVA results indicated significant differences among samples from the four regions (*R*² = 0.18, *P* = 0.001) (Fig. 3a). Similarly, for oral samples, Chifeng samples were mostly located in the first and second quadrants, Kunming samples were relatively concentrated in the first quadrant, Shanghai samples were mainly distributed around the fourth quadrant, and Urumqi samples were primarily distributed in the second and third quadrants. According to PERMANOVA, significant separation in bacterial communities was observed among oral samples from the four regions (*R*² = 0.234, *P* = 0.001) (Fig. 3b). However, for nasal bacteria, except for Urumqi samples (first and fourth quadrants), samples from the other regions were relatively scattered. The PERMANOVA results indicated significant differences among regions (*R*² = 0.09, *P* = 0.002) (Fig. 3c).

**Fig 4:**
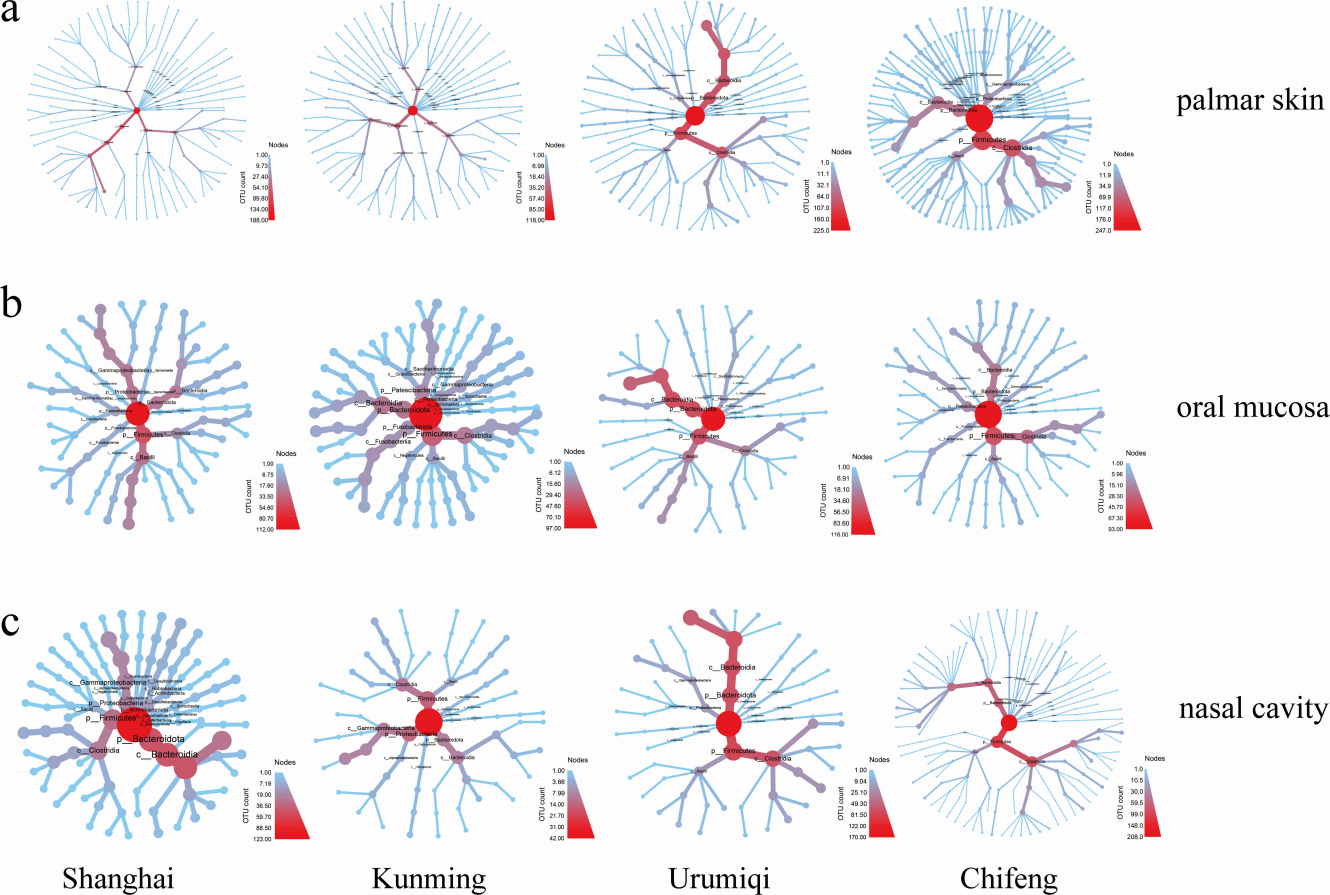
Comparative analysis of phylogenetic trees and taxonomic annotation of the (**a**) palmar skin, (**b**) oral mucosa, (**c**) nasal cavity swab samples from four regions. The nodes from the inside to the outside in the diagram represent each classification level. The color of each branch indicates the proportion of bacteria within each clade at the species level.

Additionally, we utilized OTU information to achieve a detailed taxonomic resolution of the bacterial communities at the species level. As shown in Fig. 4, the bacteria residing on palmar skin (Fig. 4a) contained more annotated species compared with those in the oral (Fig. 4b) or nasal (Fig. 4c) cavities across the four regions, as evidenced by denser circular branches in the phylogenetic tree. Moreover, for the bacteria from the same sample type, those from Chifeng showed higher branch density, suggesting greater bacterial diversity in Chifeng.

### Screening differential bacteria

**Fig 5:**
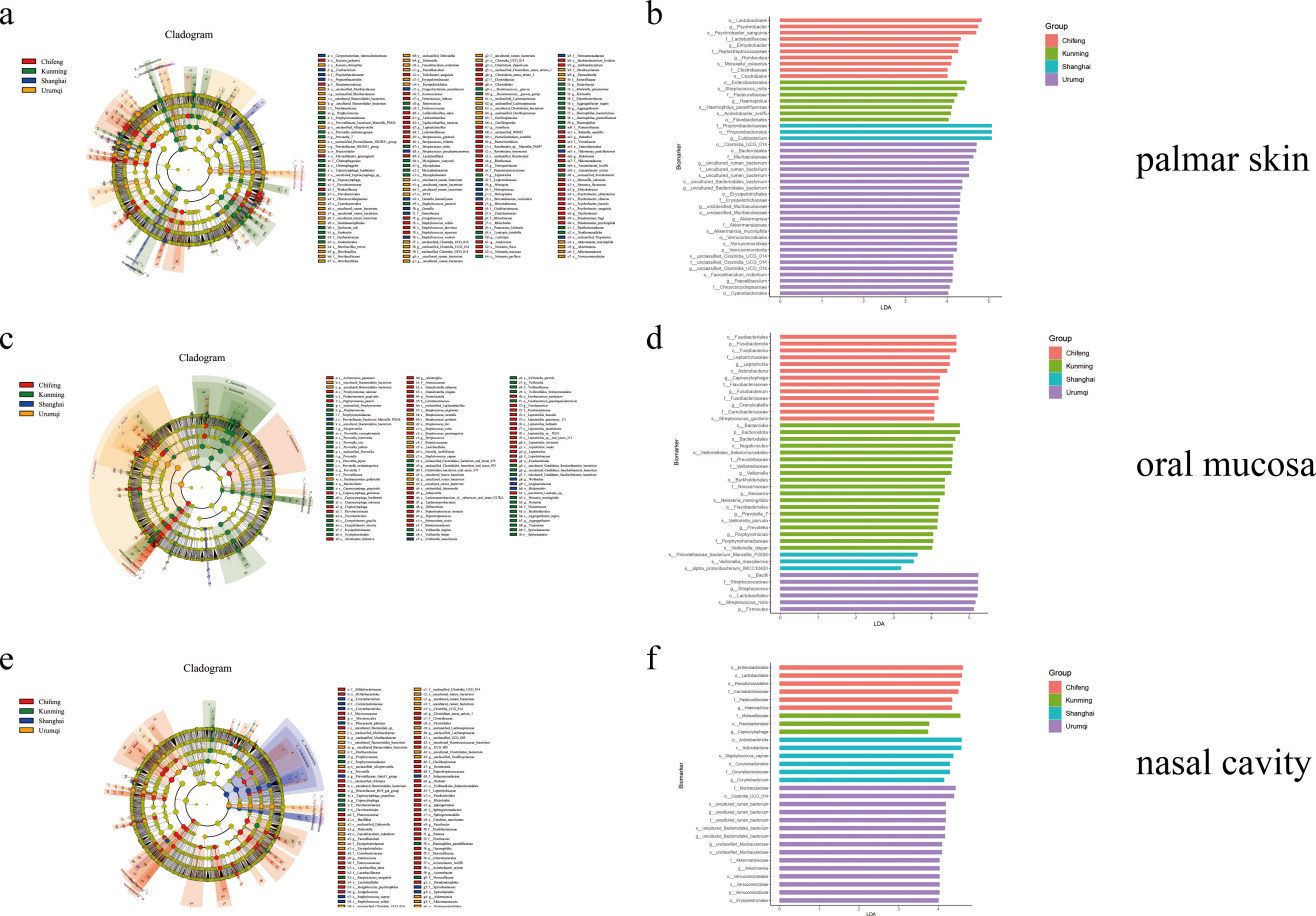
The results of the LEfSe) in the microbiome of the (**a and b**) palmar skin, (**c and d**) oral mucosa, and (**e and f**) nasal cavity from four regions. In the cladogram diagrams (a, c, e, LDA = 3), the circles from inside to outside represent the taxonomic levels from phylum to species, and the diameter of each small circle is proportional to its relative abundance. The bar graphs of LDA scores (b, d, f, LDA = 4) show the bacteria with statistically significant differences across the four regions.

Differential bacteria (from phylum to species level) of the three body sites and the four regions were selected based on linear discriminant analysis effect size (LefSe) analysis, with linear discriminant analysis (LDA) scores of 3.0 (Fig. 5a, c and e) and 4.0 (Fig. 5b, d and f). Initially, when the LDA score was 4.0, a total of 45 differential bacteria were identified from palmar skin samples. Among them, 55.56% (25) were from Urumqi, including *Clostridia_UCG_014*, *Bacteroidales, Erysipelotrichales*, and *Verrucomicrobiales* at the order level and *Muribaculaceae*, *uncultured_rumen_bacterium*, *Erysipelotrichaceae*, *Akkermansiaceae*, and *Chroococcidiopsaceae* at the family level, among others. Additionally, 10, 7, and 3 differential bacteria were observed in Chifeng, Kunming, and Shanghai, respectively (Fig. 5b). For oral samples with an LDA score of 4.0, 41 differential bacteria were identified, with Kunming contributing 46.34%, including differential bacteria at various taxonomic levels (Fig. 5d). Shanghai did not have any specific bacteria with an LDA value greater than 4.0; however, when the standard was set to 3.0, three differential bacteria at the species level (*Prevotellaceae_bacterium_Marseille_P2826*, *Veillonella_massiliensis*, and *Alpha_proteobacterium_IMCC10400*) were recognized in the Shanghai region (Fig. 5d). Similarly, a total of 30 differential bacteria were identified in nasal samples across the four regions with an LDA score of 4.0, with 50% found in Urumqi. Differential nasal bacteria for Kunming, including *Moraxelaceae*, *Flavobacteriales*, and *Capnocytophaga* at the family, order, and genus levels, respectively, were identified with an LDA standard of 3.0 (Fig. 5f).

### Geolocation prediction

First, OTUs comprising less than 0.05% of the total sample reads were filtered out. The remaining OTUs and the differential bacteria OTUs identified through LefSe screening were then divided into training and testing sets at a ratio of 7:3. The caret and Random Forest packages were utilized to predict the geolocation of the tested samples. The results showed that when all OTUs were used, the area under the curve (AUC) based on bacteria from palmar skin, oral, and nasal cavities were 0.9433, 0.9148, and 0.8883, respectively. Similarly, the AUC values based on palmar skin, oral, and nasal bacteria were 0.9442, 0.9097, and 0.8878, respectively, when using OTUs contained in bacteria with regional differences (Table 1). Additionally, geographic locations were predicted using a random forest model with the obtained genus and species, respectively. At the genus level, the AUC based on bacteria from palmar skin, oral, and nasal cavities were 0.952, 0.9432, and 0.9594, respectively; at the species level, the AUC values based on palmar skin, oral, and nasal bacteria were 0.9442, 0.9097, and 0.9158, respectively (Table S4).

**TABLE 1:** The results of the random forest model for predicting geographical location

Body site	Full-length 16S rRNA OTUs				Differential bacteria OTUs			
	Numbers	Accuracy	Kappa	AUC	Numbers	Accuracy	Kappa	AUC
Palmar skin	547	0.8421	0.7889	0.9433	249	0.8421	0.7881	0.9442
Oral mucosa	459	0.7727	0.6961	0.9148	384	0.7273	0.6343	0.9097
Nasal cavity	257	0.7619	0.6818	0.8883	120	0.7143	0.6182	0.8878

### The performance of hypervariable regions V3-V4 and V4-V5

In this study, we aimed to detect the full-length 16S rRNA gene to achieve superior taxonomic resolution and facilitate geographical inference. The V3-V4 and V4-V5 hypervariable regions of the 16S rRNA gene are commonly employed in microbiome diversity research. However, their efficacy in microbiome analyses pertaining to geographical origins remains unclear. To address this gap, we systematically compared the performance of the V3-V4 and V4-V5 hypervariable regions against full-length 16S rRNA sequences in our subsequent analyses. *In silico*, the hypervariable region sequences (V3-V4 and V4-V5) were extracted from the full-length 16S rRNA data, resulting in 24,157 (V3-V4) and 23,255 (V4-V5) OTUs, respectively. This led to the identification of 494 (V3-V4)/427 (V4-V5) species, 292/261 genera, 123/121 families, 60/65 orders, 24/27 classes, 16/19 phyla, and 1/1 kingdom. Specifically, palmar skin, oral, and nasal swab samples generated 10,506 (V3-V4)/9877 (V4-V5), 7,310/6,923, and 6,341/6,455 OTUs, respectively. The OTU distribution was as follows: 520/479, 537/486, 492/448, and 487/454 OTUs in Shanghai, Chifeng, Kunming, and Urumqi samples, respectively, with 434/402 OTUs shared among the four regions.

The investigation of microbiome composition was also performed based on V3-V4 and V4-V5 regions. At the genus level, the top 10 most abundant bacteria of palmar skin, oral, and nasal cavities in each region were identified (Fig. S2). The results indicated that in the Shanghai, Kunming, and Urumqi regions, the microbial flora of the palmar skin according to the V3-V4 or V4-V5 regions was mainly composed of *Cutibacterium*, *Staphylococcus*, *Streptococcus*, *Psychrobacter*, and others, with *Cutibacterium* being the most common bacterium (average relative abundance of 22% and 21%, respectively). In contrast, *Psychrobacter* was the dominant bacterium in the Chifeng region (average relative abundance of 14% and 13%, respectively) (Fig. S2a and d). Across the four regions, oral bacteria were predominantly composed of *Streptococcus*, *Haemophilus*, *Gemella*, *Neisseria*, and others, with *Streptococcus* being the most common bacterium (average relative abundance of 50% for both V3-V4 and V4-V5 regions) (Fig. S2b and e). Similarly, nasal bacteria were primarily comprised of *Staphylococcus*, *Cutibacterium*, *Dolosigranulum*, and others, with *Staphylococcus* being the dominant bacterium (average relative abundance of 44% and 43%, respectively) in nasal samples from all four regions (Fig. S2c and f).

The alpha diversity results based on V3-V4 and V4-V5 regions showed that for palmar and nasal bacteria, the highest alpha diversity was observed in the Chifeng group (Fig. S3a, c, d, and f), consistent with results based on full-length 16S rRNA. For oral bacteria, the highest alpha diversity was observed in the Kunming group based on V3-V4 (Fig. S3b) and in the Chifeng group based on V4-V5 (Fig. S3e), although no significant differences were found. Additionally, the PCoA results indicated that palmar and oral samples from the four regions could be geographically clustered (Fig. S4a, b, d and e), whereas nasal samples were dispersed (Fig. S4c and f), despite significant differences in the PERMANOVA results.

For predicting geographical location, OTUs from V3-V4 and V4-V5 regions were utilized to construct a random forest model. Based on the OTUs of the V3-V4 region, the palmar skin samples exhibited the highest AUC value of 0.9958 in prediction, followed by nasal and oral samples (0.9353 and 0.9312, respectively). Similarly, based on OTUs from the V4-V5 region, the AUC value of the palmar skin group remained the highest (0.9787), whereas the AUC values for the oral and nasal samples were 0.9777 and 0.9749, respectively (Table S5).

## DISCUSSION

The study analyzed the microbiota of palmar skin, nasal, and oral cavities of individuals residing in four regions of China using full-length 16S rRNA sequencing. We investigated the composition and structure of bacterial communities at these three body sites and attempted to differentiate the geographical locations of these individuals based on microbiota characteristics.

For the prominent bacteria of palmar skin, we found that the relative abundance of *Cutibacterium* was the highest in Shanghai and Kunming, consistent with published reports (22). In contrast, *Psychrobacter* and *Psychrobacillus* were the most abundant in Chifeng and Urumqi, respectively. These regions are in northern China, characterized by relatively low temperatures and dry weather. *Psychrobacter*, belonging to the Proteobacteria phylum, is a gram-negative, frost-tolerant bacterium with special physiological mechanisms and metabolic pathways to adapt to challenging environments (23). For example, the high esterifying enzyme activity of fatty acids and the high content of polyunsaturated fatty acids help maintain the fluidity and stability of cell membranes. Additionally, the synthesized antifreeze proteins protect intracellular proteins and nucleic acids from damage caused by low temperatures (24, 25). Similarly, *Psychrobacillus* can form endospores and display higher tolerance to environmental changes (26, 27), enabling them to thrive in Chifeng and Urumqi. According to Fig. 1, *uncultured_rumen_bacterium* accounted for a relatively large proportion of the Urumqi palmar group. *Uncultured_rumen_bacterium* are predominantly found in the anaerobic environment of the rumen in ruminants, such as cattle and sheep (28, 29). Urumqi, situated in the Xinjiang province, is characterized by its significant livestock farming activities, which likely result in frequent interactions between the local population with livestock. Consequently, these findings may relate to environmental factors or the lifestyle practices of the residents in this region. In contrast, *Uncultured_rumen_bacterium*, along with *Psychrobacter* and *Psychrobacillus*, showed relatively low abundance in the Shanghai and Kunming groups, ranking outside the top 10 most abundant bacteria. This indicates that these three bacteria exhibit regional preferences, as confirmed by subsequent LefSe analysis.

According to previous studies, the dominant bacteria at the genus level in the human oral microbiome are *Streptococcus*, *Neisseria*, *Prevotella*, and *Rothia* (30–33). Similar findings were observed in this study. Although *Streptococcus* was the most abundant bacterium across the four regions, its composition ratio varied, with the highest proportion found in the Urumqi oral group, followed by Shanghai, Chifeng, and Kunming. Furthermore, the bacteria ranked second in abundance differed among regions. In addition to *Streptococcus*, both *Leptotrichia* and *Haemophilus*, which are Gram-negative bacteria typically found in the oral cavity and throat as part of the normal microbial community, showed varying levels of abundance. *Leptotrichia* was the second most abundant bacterium in the Chifeng group but was relatively low in Urumqi and Shanghai. Conversely, *Haemophilus* was the second most abundant in the Shanghai and Kunming groups. These results indicate that although the primary bacteria in the oral cavity are consistent, there are regional differences in the composition and structure of bacterial communities, which could be utilized for predicting the geographic origin of oral samples.

Additionally, *Staphylococcus* was the most common nasal bacterium across all four regions, aligning with findings from previous studies (18, 34). The colonization capacity of *Staphylococcus* is likely regulated by the composition of the nasal microbiota, influenced by factors such as limited nutrients, antimicrobial molecules, epithelial inflammation, and multifactorial molecular mechanisms of nasal commensals (11). Alongside *Staphylococcus*, *Peptoniphilus*, *Dolosigranulum*, *Anaerococcus*, and *Cutibacterium* were among the top five most abundant bacteria in the nasal cavities of Chifeng, Urumqi, and Shanghai. In the Kunming group, *unclassified_Neisseriaceae* and *Streptococcus* also showed high abundance. These dominant nasal bacteria have also been revealed by previous studies (11, 35, 36). Remarkably, despite the anatomical proximity of the nasal and oral cavities, both covered by mucous membranes, the dominant bacteria differed, likely due to their distinct physiological functions and inner environments.

In the alpha diversity analysis, the results indicated that Chifeng exhibited the highest bacterial richness in both palmar skin and nasal samples, with significant differences observed when compared with other regions. In contrast, bacterial evenness remained relatively consistent across all regions. This suggested that bacterial richness could serve as a distinguishing factor for differentiating Chifeng from the other three regions. Additionally, the evenness indices for oral samples showed significant variation across the four regions, suggesting that these indices may be a valuable indicator for determining the geographic origin of the samples (Fig. 2). Subsequently, we employed Weighted_Unifrac distance and PCoA to further analyze the differences in bacterial communities among the four regions (37). For palmar bacteria, the Chifeng and Urumqi groups were distinguishable by PC2. Regarding oral bacteria, PC1 differentiated Urumqi and Kunming, whereas Chifeng and Shanghai were mainly distinguished by PC2. This suggests that dietary characteristics, climate differences, environmental exposure, and other regional factors shape oral and palmar microorganisms, consistent with previous studies (6, 38). However, the distribution of nasal samples across different regions appeared scattered, with an *R*² of 0.09 (*P* = 0.002) in the PERMANOVA, indicating that geographical factors had a minor impact on nasal bacteria. This could be due to the relatively enclosed living environment of nasal microorganisms. Although nasal microbes may not be as prominent as palmar skin and oral microbes in determining geographic origin, and few studies have incorporated nasal microbes from distinct locations, this study investigates their characteristics and efficiency, providing a valuable reference for subsequent research.

Based on the linear discriminant analysis of different regional samples by taxonomy, LefSe analysis identified bacteria with significant differences in the three body sites and across the four regions. Urumqi had the most differential bacteria on the palmar skin (23), whereas Kunming had the most oral-specific bacteria (19). Shanghai had the least number of differential bacteria in these two sample types, illustrating a high overlap in bacterial structure between Shanghai and the other three regions, making it more challenging to predict unknown samples from Shanghai. This result was expected, as Shanghai is a major city with high mobility, attracting people from all over China with diverse genetic backgrounds, diets, and lifestyles, resulting in fewer specific bacteria compared with people in other areas.

Random forest is one of the most widely used machine learning algorithms and was employed to construct a geographical prediction model using all bacterial OTUs and OTUs from differential bacteria, respectively. Whether based on all OTUs or differential OTUs, the performance of using palmar bacteria to predict geographic location was superior to that of using nasal and oral cavity bacteria. This result could be attributed to the fact that palmar skin is exposed to the external environment and is easily affected (39), whereas environmental factors such as temperature and humidity are relatively stable in the nasal and oral cavities (40), resulting in smaller regional differences. Although the difference was not significant (*P* = 0.478, *t*-test), the performance of the prediction model constructed using all OTUs was slightly higher than that of using OTUs from differential bacteria. This is likely due to a larger number of features providing more taxonomic information. However, more features can increase the complexity of the prediction model and may also lead to overfitting. The prediction model constructed using the differential bacteria OTUs had a certain loss in accuracy, but it greatly reduced the number of features used. Moreover, when we predicted geographic location using obtained genus and species information, higher AUC values were observed (*P* > 0.05). This suggests that discovering more suitable features and machine learning methods is essential to achieve better prediction results in practice.

Several previous studies have demonstrated that different hypervariable regions of the 16S rRNA gene provide varying degrees of resolution in the identification of taxonomic groups, leading to different estimates of the composition of microbial communities (41–43). Typically, microbiome diversity is analyzed based on the V3-V4 and V4-V5 hypervariable regions of 16S rRNA (44). In this study, we compared the performance of V3-V4, V4-V5, and full-length 16S rRNA sequences in bacterial composition analysis and geographical origin differentiation. The results indicated that compared with V3-V4 and V4-V5, more OTUs (an average increase of 157.96%) and more bacteria (an average increase of 315.01%) at the species level were identified using full-length 16S rRNA data. Subsequently, in the analysis of microbiome composition, the top 10 most abundant genus-level bacteria of the three body sites in each region showed the greatest similarity in the oral cavity group with those based on full-length 16S rRNA. In the palmar skin and nasal cavity groups, although the top several abundant bacterial genera also showed strong similarity, slight differences were observed in other bacterial genera or their proportions. For example, *Klebsiella* was not found among the top 10 bacterial genera residing in the nasal cavity based on V4-V5 OTUs, whereas it ranked as the sixth and ninth most abundant genus, respectively, using the OTUs of full-length 16S rRNA and V3-V4 regions. Interestingly, the ROC analysis indicated that the random forest model had comparable performance (*P* > 0.05) in distinguishing the four regions based on V3-V4 (AUC > 0.95), V4-V5 (AUC > 0.97), and full-length 16S rRNA (AUC > 0.91), suggesting that machine learning models exhibited varying degrees of adaptability when handling different data (45, 46).

This study preliminarily explored the potential of using the microbiome of the palmar skin, oral, and nasal cavities for predicting geographic origin. However, machine learning algorithms typically require large samples to support the reliability of the results. In the future, by expanding the sample size, the diversity and overall characteristics of data could be better revealed, thereby improving the accuracy and reliability of model predictions.

### Conclusion

The study investigated and characterized the microbiota of the palmar skin, oral, and nasal cavities of Han individuals from four distinct regions in China, utilizing single-molecule real-time sequencing of the full-length 16S rRNA gene. The results revealed comprehensive insights into the composition and structure of the microbiomes at these three body sites, highlighting significant regional differences. Additionally, this study demonstrated the substantial potential of the human microbiome for forensic geographical inference, with the palmar skin microbiome exhibiting the highest predictive accuracy.

## MATERIALS AND METHODS

### Sample collection

**Fig 6:**
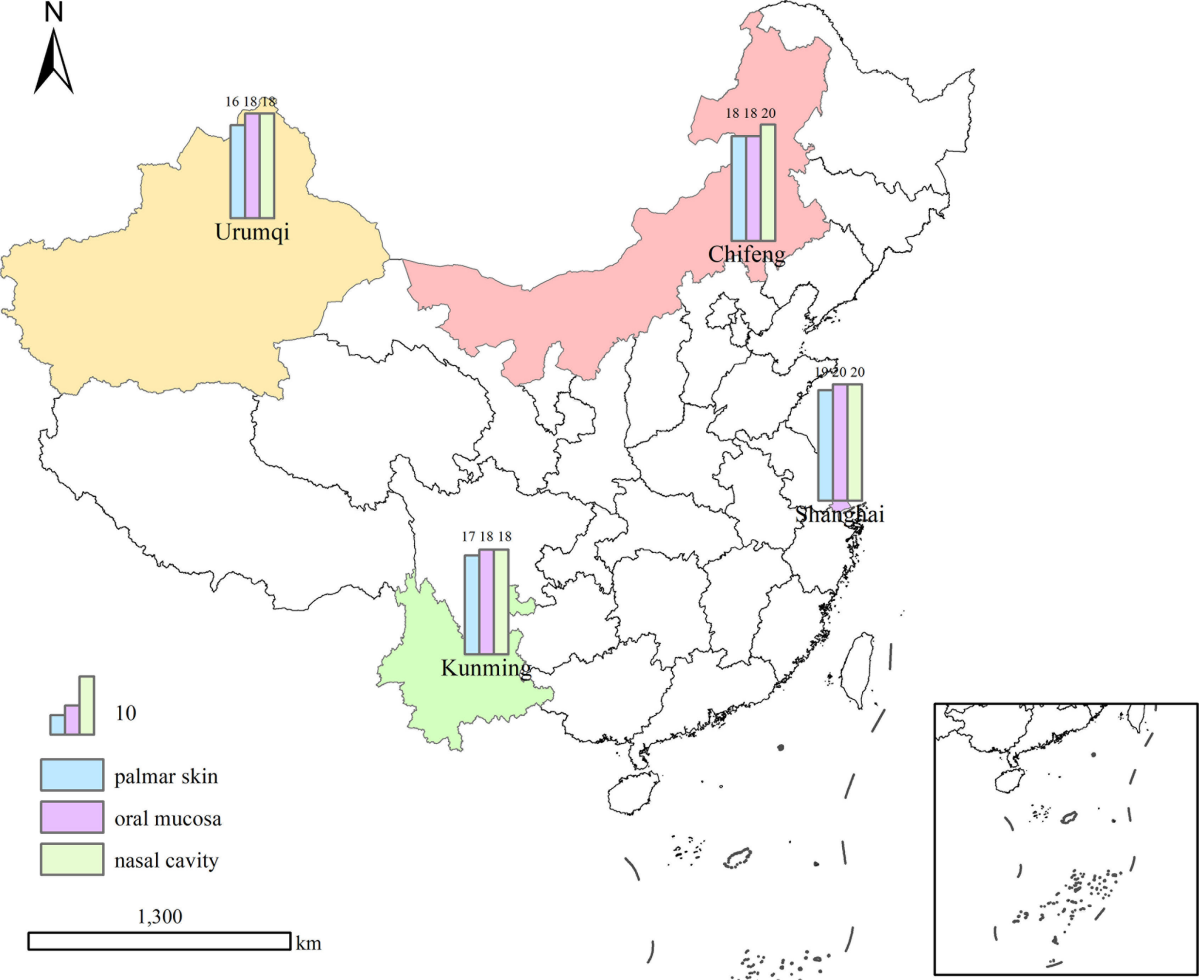
Map of four major cities in China where samples were collected. The number of samples in each site is shown in the diagram. The figure was re-generated using ArcGIS software based on the Standard Map of China, which was downloaded from the official website of Tianditu (https://cloudcenter.tianditu.gov.cn/administrativeDivision), with the map review number GS(2024)0650.

A total of 220 microbial samples were collected from the Han population in Shanghai, Chifeng, Kunming, and Urumqi, China (Fig. 6). The inclusion criteria for participants were as follows: (i) Han population without significant oral or skin diseases, such as dental caries, periodontitis, acne, or other conditions that could potentially influence the outcomes; (ii) none of the participants traveled out of their long-term residential area within 3 months before sampling; and (iii) no antibiotic treatment within 3 months before sampling. To ensure a more accurate representation of each area, sample selection was balanced in terms of gender (male-to-female ratio of 1:1) and age (20 to 50 years old) wherever possible. To protect the privacy of the volunteers, all samples were anonymized, as detailed in Table S6. All subjects provided informed consent.

Participants were required to refrain from washing their hands and consuming food or drink for a period of 2 hours prior to sampling. Oral samples were collected from the buccal mucosa using sterile swabs. Nasal samples were obtained using a sterile swab moistened with ST solution (0.15 M NaCl, 0.1% Tween 20), which was inserted 1 cm into the nasal cavity, then rotated and wiped. Palmar skin samples were collected using a sterile swab moistened with ST solution, vigorously swabbing the entire surface of the palm of the dominant hand. Negative controls were sterile swabs moistened with ST solution. All samples were stored at −80°C prior to DNA extraction.

### DNA extraction, PCR amplification, and sequencing

DNA was extracted from all samples using the PowerSoil DNA Isolation Kit (QIAGEN, Germany) following the manufacturer’s instructions. PCR amplification was performed using the universal 27F (5′-AGRGTTTGATYNTGGCTCAG-3′) / 1492R (5′-TASGGHTACCTTGTTASGACTT-3′) full-length primers for the 16S rRNA gene, labeled with a barcode. The amplification system consisted of 1.5 µL DNA template, 3 µL Primer Mix, 15 µL KOD One PCR Master Mix, and 10.5 µL enzyme-free water. The fragment size of the amplified library for each sample was detected by 1.8% agarose gel electrophoresis. The concentration of the amplified library was measured using a Qubit 2.0 fluorometer, and the libraries were then mixed proportionally. Finally, the SMRT-Bell Preparation Kit 3.0 (PacBio, USA) was used to construct the template-primer-polymerase sequencing complex, which was subsequently sequenced on the PacBio Sequel II platform.

### Statistical analysis

The original CCS data were identified through the barcode using lima v1.7.0 software. Circular consensus sequence (CCS) reads were obtained with the following settings: minimum number of passes = 3 and minimum predicted accuracy = 0.99. Primer sequences were removed using Cutadapt v1.9.1, and CCS sequences were filtered to maintain sequence lengths in the range of 1,200 bp–1,650 bp (47). Chimeras were removed using UCHIME v4.2 software, and the resulting OTUs were clustered at 97% similarity using Usearch software (http://drive5.com/uparse/) (48). Feature sequences were taxonomically annotated using a naive Bayesian classifier combined with a comparison against the reference database (https://www.arb-silva.de/). Alpha diversity was assessed by the Simpson, Chao 1, Shannon, and ACE index, calculated using QIIME2, followed by the Wilcoxon signed-rank test to detect differences between groups. Beta diversity was visualized using principal coordinates analysis (PCoA) based on the weighted_unifrac distance matrix, and differences in bacterial communities were assessed using permutational multivariate analysis of variance (PERMANOVA). LEfSe was used for multilevel microbial discrimination analysis across the four locations, and a random forest prediction model was constructed to evaluate geographic predictive power based on OTUs. The area under the ROC curve (AUC) was calculated to assess the performance of the predictive model. All statistical analyses and visualizations were performed using R software (https://www.r-project.org/) unless otherwise stated.

## Data Availability

The original contributions presented in the study are included in the text and supplemental material; further inquiries can be directed to the corresponding authors.

## SUPPLEMENTAL MATERIAL

Supplemental figures msphere.00672-24-s0001.docx Figures S1-S4.

Supplemental tables msphere.00672-24-s0002.xlsx Tables S1-S6.

ASM does not own the copyrights to Supplemental Material that may be linked to, or accessed through, an article. The authors have granted ASM a non-exclusive, world-wide license to publish the Supplemental Material files. Please contact the corresponding author directly for reuse.
