# Supplementary material for: Comparative analysis of the human microbiome from four different regions of China and machine learning-based geographical inference
Source: mSphere. 2024 Dec 19;10(1):e00672-24. doi: 10.1128/msphere.00672-24 (PMC11774049; doi:10.1128/msphere.00672-24)
Supplement: Supplemental figures — Figures S1-S4. [file msphere.00672-24-s0001.docx]

Fig. S1 Bacterial community composition of the (a) palmar skin, (b) oral mucosa, and (c) nasal cavity swab samples from four regions at the species level.





Fig. S2 Bubble map of the bacterial community composition at the genus level in the (a, d) palmar skin, (b, e) oral mucosa and (c, f) nasal cavity swab samples from four regions, based on (a, b, c) V3-V4 and (d, e, f) V4-V5 regions.


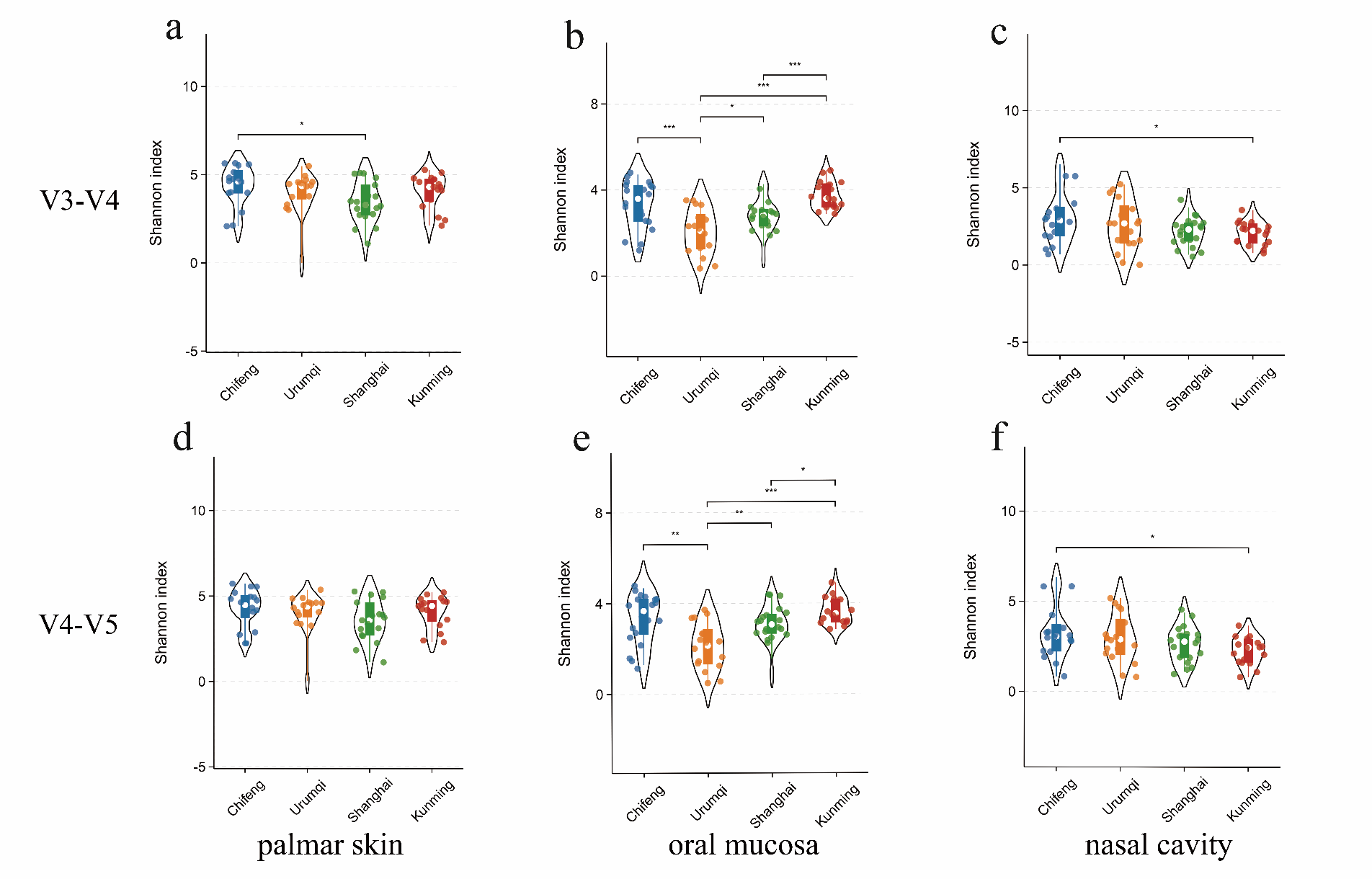


Fig. S3 Shannon’s diversity index of the (a, d) palmar skin, (b, e) oral mucosa and (c, f) nasal cavity swab samples from four regions, based on (a, b, c) V3-V4 and (d, e, f) V4-V5 regions (***, p＜0.001; **, p＜0.01; *, p＜0.05).


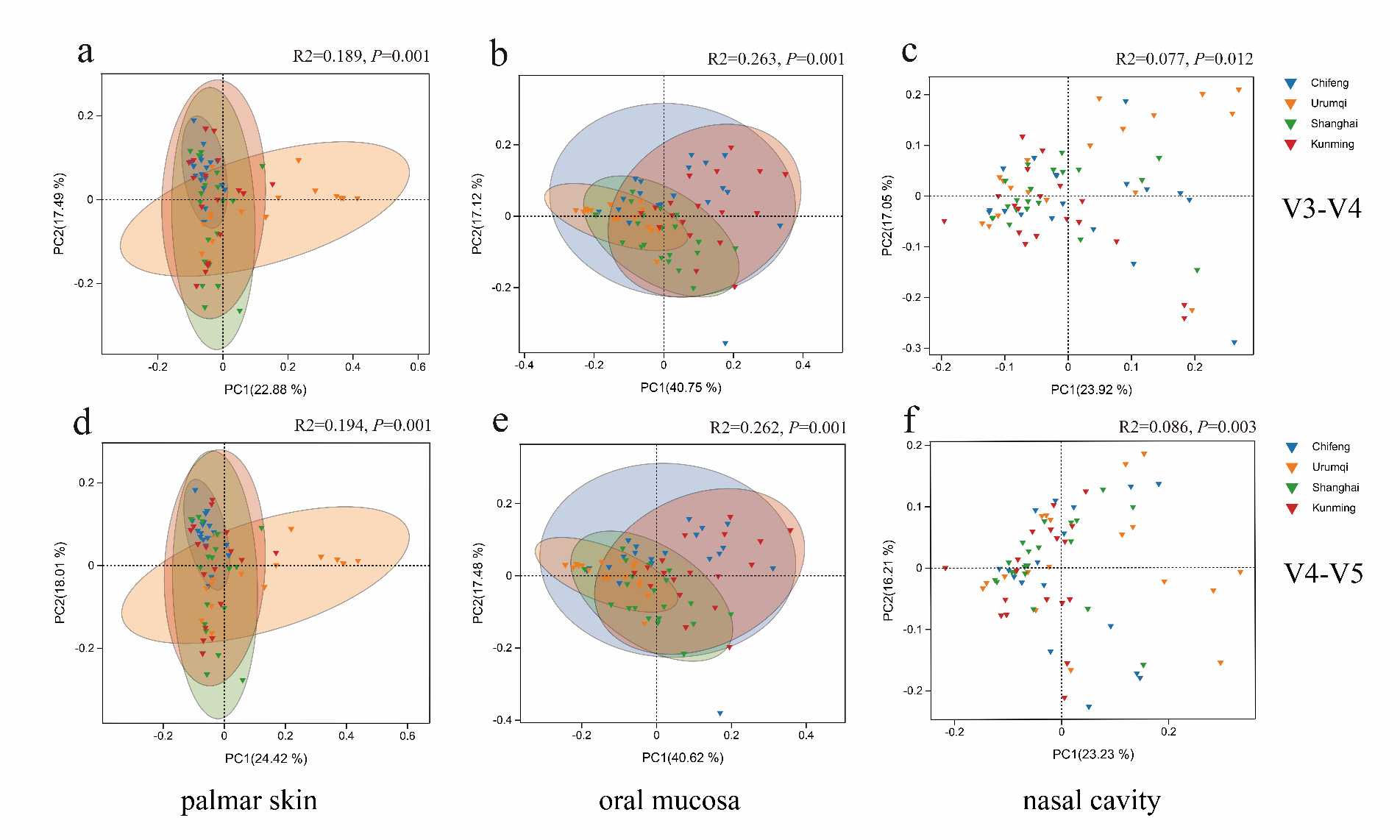


Fig. S4 Principal coordinates analysis (PCoA) of the (a, d) palmar skin, (b, e) oral mucosa and (c, f) nasal cavity swab samples from four regions, based on (a, b, c) V3-V4 and (d, e, f) V4-V5 regions.
